# Practical starter pig amino acid requirements in relation to immunity, gut health and growth performance

**DOI:** 10.1186/2049-1891-5-12

**Published:** 2014-02-18

**Authors:** Bob Goodband, Mike Tokach, Steve Dritz, Joel DeRouchey, Jason Woodworth

**Affiliations:** 1 Department of Animal Sciences and Industry, Kansas State University, Manhattan, KS 66506-0201, USA; 2 Department of Diagnostic Medicine/Pathobiology, College of Veterinary Medicine, Kansas State University, Manhattan, KS 66506-0201, USA

**Keywords:** Amino acids, Immunity, Pigs, Requirements

## Abstract

Immune system activation begins a host of physiological responses. Infectious agents are recognized by monocytes and macrophages which in turn stimulate cytokine production. It is the hormone-like factors called cytokines that orchestrate the immune response. The classic responses observed with immune system activation and cytokine production include: anorexia, fever, lethargy, recruitment of other immune cells, and phagocytosis. While production of immune system components is known to require some amino acids, increases in amino acid requirements are more than offset by the associated decrease in protein accretion and increased muscle protein degradation that also accompanies immune system activation. However, the biggest impact of cytokine production is a decrease in feed intake. Therefore, as feed intake decreases, the energy needed to drive protein synthesis is also decreased. This suggests that diets should still be formulated on a similar calorie:lysine ratio as those formulated for non-immune challenged pigs. The evidence is sparse or equivocal for increasing nutrient requirements during an immune challenge. Nutritionists and swine producers should resist the pressure to alter the diet, limit feed, or add expensive feed additives during an immune challenge. While immune stimulation does not necessitate changes in diet formulation, when pigs are challenged with non-pathogenic diarrhea there are potential advantages on gut health with the increased use of crystalline amino acids rather than intact protein sources (i.e., soybean meal). This is because reducing crude protein decreases the quantity of fermentable protein entering the large intestine, which lowers post weaning diarrhea. It also lowers the requirement for expensive specialty protein sources or other protein sources such as soybean meal that present immunological challenges to the gut. The objective of this review is two-fold. The first is to discuss immunity by nutrition interactions, or lack thereof, and secondly, to review amino acid requirement estimates for nursery pigs.

## Introduction

World-wide swine production has evolved dramatically in the last decade. Genetic improvements have dramatically increased reproductive traits such as litter size as well as improve growth traits like daily gain and feed efficiency. Multiple site production has made a large impact on herd health and weaned pig flow management. Practical nutrition programs continue evolving to keep pace with these rapid changes and to improve profitability of pork producers. One important concept that has risen from these changes is the interaction of nutrition and immunity or herd health. Feeding pigs based on their immune status or pathogen challenge was once a novel idea based on feeding specifically formulated diets to meet the different amino acid requirements of the immune system. While studies have observed that up-regulation of the immune system may slightly impact amino acid requirements for leukocyte and cytokine production [1], the major driver of a nutrition/immune response interaction resides in the response to an immune challenge of lower feed intake and in some cases poorer feed efficiency [2-4]. Therefore, because of decreased energy (feed) intake, the body will most likely not support excess amino acid supplementation for protein synthesis to combat the effects of immune system activation [5]. In addition, from an enteric pathogen stress point of view, recent studies have observed that practices to minimize post weaning scours, such as restrictive feeding of pigs at weaning or providing high-fiber diets will contribute to the decreased growth performance in the nursery stage [6,7]. Therefore, these data suggest that from a practical feeding standpoint, there is no interaction between immune challenge and diet complexity on pig performance which indicates that relatively high complexity diets containing specialty protein sources are just as valuable for healthy pigs as those faced with an immune challenge [5]. Thus, producers should avoid reformulating diets if environmental conditions are less than ideal. Ultimately, by maintaining a relatively high amount of specialty protein sources and utilizing the proper amino acid ratios with crystalline amino acid supplementation, dietary crude protein can be lowered and excellent growth performance in the nursery can be maintained.

### Nutrition by immune system activation interactions

To evaluate immune system activation by nutrition interactions in pigs, Williams et al. [2,3] observed that the efficiency of lysine utilization for protein deposition was similar among pigs with high or low immune system activation. Thus, differences in feed efficiency among challenge groups could be explained by shifts in ratios of lean and fat deposition and proportion for maintenance. This indicates that healthy pigs with relatively low immune system activation have greater need for dietary lysine as a consequence of greater protein deposition compared to those pigs with high immune system activation. Increasing pathogen load stimulated pro-inflammatory cytokine production and endocrine shifts which not only decreased feed intake, but increased muscle catabolism [1]. The high immune stimulated pigs have less protein deposition, hence less energy needed to drive body protein accretion. Decreasing pathogen load and thus lowering immune stimulation resulted in greater feed intake and growth performance [2,3]. More recently, research evaluating PCV2 vaccination under commercial conditions indicated that vaccinated pigs had a greater need for lysine on a grams per day basis [5]. However, in this study when evaluated on a lysine to calorie ratio, the requirement was not different between vaccinated and unvaccinated pigs even though there were large differences in growth performance and mortality rates. From a practical standpoint this would support the idea that a similar calorie:lysine ratio should be maintained regardless of immune system activation status, or in other words diet modifications are not warranted when the immune system is activated.

It is possible that immune system activation will affect the utilization of some amino acids relative to lysine. However data to support this effect is difficult to find. Methionine is probably the most studied amino acid other than lysine in response to an immune challenge. Naturally diets deficient in essential amino acids like lysine or methionine, will not support cytokine (IL-1) production and will further reduce growth beyond that observed due to decreased feed intake [8]. Again, decreased muscle protein accretion and increased degradation appear to offset shifts in immune-related protein synthesis.

It is important to recognize that viral, bacterial, or mixed pathogen challenges may elicit different immune responses. However, from the chronic mixed challenge model used by Williams et al. [1,2] to the more acute, viral challenge of Shelton [5]; responses in protein deposition and feed intake were similar. However more research in this area is necessary to determine if other types of immune challenge may have differing effects on feed intake.

**Figure 1:**
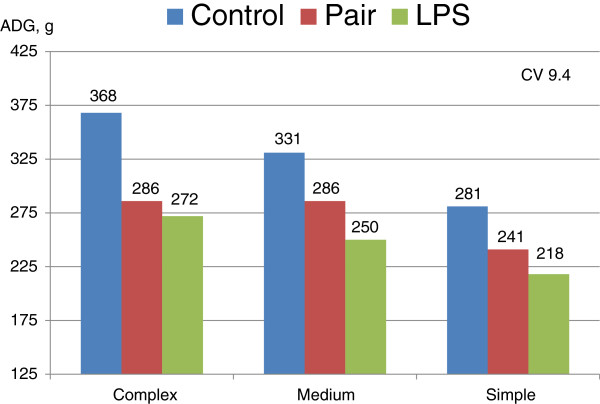
**The effects of immune system activation and diet complexity on average daily gain.** Dietary treatments include feeding a complex starter diet, a medium complexity diet, and a low complexity diet. Immune activation includes control pigs fed ad libitum, LPS injected pigs fed ad libitum, and control pigs pair-fed to that of the LPS challenged pigs. Main effects of diet complexity and immune system activation are significant (*P* < 0.01). There was no immune system by diet complexity interactions (Adapted from Dritz et al., 1996 [4]).

As it appears that there is little need to adjust diets based on immune system activation in growing-finishing pigs, Dritz et al. [4] evaluated the interactive effects of a lipopolysaccharide (LPS) induced immune challenge and diet complexity on weanling pig performance. In this study the three comparisons consisted of control pigs fed ad libitum, pigs challenged with LPS and fed ad libitum, or non-challenged pigs pair fed to the same feed intake level of the LPS pigs. In addition, there were 3 diet complexity regimens used: a complex diet using high amounts of specialty protein sources (animal blood plasma, fish meal, blood meal, and dried whey), intermediate amounts of these specialty ingredients, and then a very simple diet with minimal amounts of these ingredients. The LPS challenged pigs had increased haptoglobin concentrations indicating the inflammatory cytokine production was increased in immune challenged pigs. Control pigs had increased ADG and were heavier at the end of the study, whereas LPS challenged pigs were the lightest, with pair fed pigs intermediate (Figure 1). There were no immune status × diet complexity interactions observed suggesting that the response to immune challenge is independent from diet complexity. That is, pigs fed the complex diet regimen had the greatest ADG regardless of immune system activation or pair feeding. Pigs administered LPS had poorer performance than those that were pair fed resulting from a combination of reduced ADFI and poor G:F. The intermediate performance of the pair-fed pigs suggests that approximately 2/3 of the reduction in growth was feed intake related, whereas 1/3 was due to poorer G:F. Ultimately this study confirms that the diets fed to pigs in an immune challenged environment should be similar to that of pigs fed in a clean environment.

More recently, the effects of an immune challenge as a result of housing weanling pigs in either “clean” or “dirty” environments has been addressed by Montagne et al. [6]. To potentially reduce the incidence of post weaning diarrhea, one hypothesis is that feeding a diet high in fermentable fiber might increase the population of beneficial bacteria. This is thought to alleviate the effects of pathogenic bacteria by competitive exclusion for binding sites within the gut. In this study, weanling pigs were housed in either a clean (washed and disinfected) nursery or one that had not been cleaned after the previous group of pigs [6]. They were either fed a control diet or high fiber diet (d 0 to 14: 3.25 or 4.89% crude fiber and 12.1 or 16.9% total dietary fiber, respectively). There were no environment × diet interactions, and pigs in the dirty environment had poorer G:F than those in the clean environment (Table 1). The addition of fiber to the diet decreased NE intake and tended to decrease ADG and ADFI. Pigs housed in the dirty environment and fed the high fiber diet were 0.50 kg lighter than counterparts fed the control diet after 1 wk. The authors confirmed that poor sanitary conditions reduced pig growth and increased the incidence of digestive disorders in the first week post-weaning. Feeding a high fiber diet to pigs housed in a dirty environment further decreased growth [6]. Rather than being beneficial, the addition of fiber reduced performance in both the clean and dirty environment.

**Table 1:** **Effects of added dietary fiber in either a clean or dirty environment on weanling pig performance (Montagne et al.**[6]**)**^**1**^

**Items**	**Clean**		**Dirty**	
	**Control**	**Fiber**	**Control**	**Fiber**
d 0 to 14				
ADG, g^2^	128	127	132	91
ADFI, g^2^	228	218	275	241
G:F^3^	.524	.543	.452	.424

^1^Pigs assigned to the good sanitary conditions were housed in cleaned and disinfected rooms; pigs assigned to the poor sanitary conditions were housed in rooms that were not cleaned; the Control and Fiber diets used during d 0 to 14 were 121 and 169 g/kg of total dietary fiber, respectively.

^2^Effect of added dietary fiber, (*P* < 0.10).

^3^Effect of sanitary condition, (*P* < 0.01).

Recently, other options to reduce the risk of post-weaning diarrhea evaluated restricted vs.ad libitum feeding immediately post weaning [7]. Similar to the previous paper [6], pigs were housed in either a clean or dirty environment but in this study they were either fed ad libitum or a restrictive feeding regimen from day 2 to 7 after weaning. Again, no environment × feeding regimen interactions were observed indicating that the response to each was independent (Table 2). The authors concluded that feed restriction immediately after weaning exacerbated the effects of poor sanitary conditions [7].

**Table 2:** **Effects of restrictive feeding in either a clean or dirty environment on weanling pig performance (Pastorelli et al. **[7]**)**^**1**^

**Items**	**Clean**		**Dirty**	
	**Ad libitum**	**Restricted**	**Ad libitum**	**Restricted**
d 0 to 11				
ADG, g^2,3^	257	159	173	.95
ADFI, g^3^	336	219	319	225
G:F^2,4^	.753	.729	.537	.393
Overall (d 0 to 60)				
ADG, g^5^	511	492	463	394
ADFI, g	875	821	826	705
G:F^2^	.587	.599	.562	.555

^1^Pigs assigned to the good sanitary conditions were housed in cleaned and disinfected rooms and received an antibiotic supplementation; pigs assigned to the poor sanitation conditions were housed in rooms that were not cleaned; the ad libitum group corresponded to pigs nourished ad libitum on overall experimental period; the restricted group corresponded to pigs that received, from 2 to 7 d after weaning, respectively, 20, 30, 40, 60, 80, and 90% of the amounts of feed voluntary consumed by ad libitum pigs in both sanitary conditions at each previous day. No feed restriction × sanitary conditions interactions, (*P* > 0.10).

^2^Effect of sanitary condition, (*P* < 0.01).

^3^Effect of feed restriction, (*P* < 0.01).

^4^Effect of feed restriction, (*P* < 0.07).

^5^Effect of sanitary condition, (*P* < 0.10).

Results of these 4 studies indicate that when faced with a disease challenge, weanling pigs need a high quality diet, but not one different than what would be provided to pigs with minimal disease challenge.

**Figure 2:**
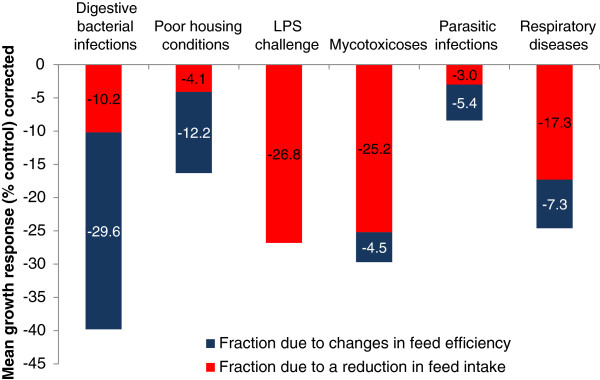
**Metabolic consequences of an activated immune system.** Partitioning the percentage decrease in average daily gain and feed efficiency as a result of different immune challenges (adapted from Pastorelli et al. [9]).

In an excellent meta-analysis covering 121 different studies, Pastorelli et al. [9] examined the effects of an immune system challenge on feed intake and growth responses. They examined the performance responses to digestive bacterial infections, sanitary housing conditions, LPS challenge, mycotoxicoses, parasitic infections and respiratory disease. They established the percentage change in growth as a result of poorer G:F or reduced daily feed intake (Figure 2). Digestive bacterial infections had the greatest negative impact on growth responses with approximately 2/3 related to poorer G:F and 1/3 related to poorer feed intake.

Like the response to digestive bacterial infection, unsanitary housing conditions resulted in the majority of the decreased performance as a result of poorer G:F, whereas LPS challenge, mycotoxicosis, and respiratory disease were almost solely feed intake driven. Again, while there may be transient changes in amino acid requirements for maintaining the immune system, decreased feed intake and poorer G:F suggest that highly digestible diets with ingredients that stimulate feed intake seem to be the best course of action in getting pigs through an immune system challenge. However, the method of supplying these amino acids in properly formulated low-protein, amino acid fortified diets may be one option to reduce post-weaning diarrhea.

### Minimizing nutritional challenges to the gut - low-protein, amino acid fortified diets

One method to decrease the dietary challenge imposed on the gastrointestinal system is to lower the dietary crude protein level. However, it is crucial to emphasize that although these diets are lower in crude protein compared with traditional formulations; they still meet amino acid requirements and support excellent pig growth performance. Reducing the crude protein content lowers the requirement for protein ingredients, such as soybean meal, that present immunological challenges to the gut as well as decreases inclusion of expensive specialty protein sources. Presenting the large intestine with a large quantity of undigested nitrogen appears to be a factor in post weaning diarrhea [10]. Lowering the quantity of protein in the diet decreases the ammonia concentration in the small intestine and urea nitrogen and volatile fatty acids in the ileum [11]. It is thought that the decreased nitrogen concentrations are due to reduced protein fermentation by the bacteria [12].

In summary, these studies would suggest that major changes in diet formulations offered to pigs during immune system activation are not warranted. The only considerations would be to ensure that crude protein is not overfed by using the optimum levels of crystalline amino acids which helps prevent large amounts of undigested nitrogen being present in the large intestine and thus contributing to diarrhea. Because changes to dietary amino acid concentrations on a lysine to calorie ratio basis are not needed during immune system activation, the remainder of this review will focus on defining the amino acid levels for weanling pig diets.

### Lysine requirements for weanling pigs

Numerous trials have explored the SID lysine requirement of nursery pigs in recent years and requirement estimates have been established (Table 3). The requirement estimate for the 5 to 10 kg pig was found to be between 1.35 and 1.40% standardized ileal digestible (SID) lysine (4.0 to 4.2 g/Mcal ME [13]. This requirement was similar to the estimate found by Dean et al. [14] of 1.40% SID lysine or 18.9 g of SID lysine per kg of gain for 6 to 12 kg pigs.

**Table 3:** **Effects of lysine level fed during each phase on nursery pig performance**^**a**^

**Items**	**Standardized ileal digestible lysine, %**											
d 0 to 7	1.35	1.35	1.35	1.35	1.55	1.55	1.55	1.55				
d 7 to 21	1.15	1.15	1.35	1.35	1.15	1.15	1.35	1.35	Probability, *P* <			
d 21 to 35	1.05	1.25	1.05	1.25	1.05	1.25	1.05	1.25	SEM	Phase 1	Phase 2	Phase 3
d 0 to 7												
ADG, g	161	151	152	162	155	163	159	161	19.9	0.69	0.89	0.72
ADFI, g	171	164	157	164	145	150	149	162	15.0	0.37	0.94	0.55
G:F	0.962	0.926	0.965	0.997	1.054	1.089	1.074	0.984	0.059	0.01	0.93	0.63
d 7 to 21												
ADG, g	363	365	366	371	346	333	370	375	15.8	0.41	0.18	0.98
ADFI, g	541	530	512	521	508	506	498	517	18.4	0.16	0.49	0.78
G:F	0.674	0.687	0.716	0.711	0.680	0.660	0.742	0.723	0.016	0.75	0.03	0.43
d 21 to 35												
ADG, g	561	616	579	614	555	573	540	593	35.1	0.20	0.78	0.001
ADFI, g	934	915	943	956	907	883	883	925	34.6	0.37	0.53	0.85
G:F	0.601	0.674	0.614	0.643	0.613	0.649	0.612	0.640	0.031	0.60	0.39	<.0001
d 0 to 35												
ADG, g	402	422	406	426	389	395	395	419	11.3	0.15	0.30	0.03
ADFI, g	745	726	730	747	711	701	696	732	20.5	0.38	0.74	0.65
G:F	0.645	0.692	0.666	0.683	0.658	0.676	0.681	0.688	0.011	0.52	0.07	0.001
BW, kg												
d 0	5.71	5.70	5.73	5.68	5.71	5.75	5.71	5.71	0.05	0.59	0.24	0.43
d 7	6.84	6.76	6.79	6.81	6.80	6.89	6.83	6.83	0.19	0.67	0.91	0.85
d 21	11.93	11.86	11.95	12.00	11.67	11.55	12.01	12.09	0.32	0.54	0.14	0.94
d 35	19.78	20.64	20.05	20.59	19.44	19.57	19.57	20.38	0.36	0.14	0.37	0.04

^a^A total of 320 weanling pigs (PIC 1050 barrows, initially 5.71 ± 0.05 kg and 21 d of age) were used in a 35-d trial with 8 pens per treatment. Phase 1, 2, and 3 diets were fed from d 0 to 7 (SID Lys 1.35 vs 1.55%), 7 to 21 (SID Lys 1.15 vs 1.35%), and 21 to 35 (SID Lys 1.45 vs 1.25%) after weaning, respectively. There were no interactions among the different phases. Nemecheck et al. [19].

For 10 to 25 kg pigs, Kendall et al. [15] conducted 5 experiments with 3,628 pigs and found the SID lysine requirement to be 1.30% SID lysine (3.80 g/Mcal ME). This was equivalent to 19 g of SID lysine per kg of gain. Schneider et al. [16] evaluated energy and lysine levels simultaneously in two separate trials with different genotypes. With one genotype, the optimal SID lyine:ME ratio was 3.4 to 3.6 g/Mcal ME, while the optimal ratio was 3.9 to 4.2 g/Mcal ME for the other genotype. However, when expressed relative to gain, the requirement was approximately 19.0 g of SID lysine/kg of gain for both genotypes. In another large field study, Lenehan et al. [17] found the SID lysine requirement for 10 to 20 kg pigs was 1.40%; and when calculated on a g/kg of gain basis, the optimal level was again 19 g of SID lysine/kg of gain.

Although lysine requirements of nursery pigs have increased in recent years and vary with environmental conditions and genotype, when expressed relative to growth rate, empirical studies have consistently found the requirement to be 19 g per kg of gain.

While historically diets for early weaned pigs (4.5 to 5.5 kg) have been formulated to 1.65 or 1.70% total lysine (1.55 to 1.65% SID lysine) or greater, Nemecheck et al. [18] observed that slightly lower dietary lysine levels can be fed in the early nursery phases without negative impact on overall ADG or BW, as long as diets during the late nursery period are adequate in lysine (Table 3). In this study, there were a total of 8 dietary treatments arranged in a 2 × 2 × 2 factorial. During phase 1 (d 0 to 7), pigs were fed diets containing either 1.35 or 1.55% SID lysine, followed by either 1.15 or 1.35% SID lysine in phase 2 (d 7 to 21), and 1.05 or 1.25% SID lysine during phase 3 (d 21 to 35). The low dietary lysine concentrations were achieved by reducing both crystalline lysine and a portion of the intact protein sources from the high lysine diets. From d 0 to 7, there were no differences in ADG or ADFI but increasing SID lysine improved G:F. Similar to phase 1, from d 7 to 21, there were no differences in ADG or ADFI between pigs fed the two lysine levels, but increasing SID lysine improved (*P* < 0.03) G:F. During phase 3, feeding the high lysine diet increased ADG and G:F, but had no effect on ADFI. For the overall trial (d 0 to 35), pigs fed the high lysine during phase 3 had the greatest improvement in ADG and G:F. There were no interactions between phases, which indicate that the response to lysine in one phase is not influenced by the lysine level fed in other phases. This allows for formulation of lower lysine (and thus crude protein) diets in early nursery phases and could result in an economical advantage by reducing feed costs while maintaining optimal growth performance.

Until recently, lowering the crude protein level in the diet usually corresponded with reduced growth performance because the minimum requirement for the fourth, fifth, or sixth amino acids (often tryptophan, valine, or isoleucine) or nonessential amino acids that have a role in gut development (arginine, glutamine, or glycine) were not met. However, numerous recent research trials have demonstrated that performance can be maintained when the crude protein level in the diet is reduced by using crystalline amino acids to replace intact protein sources [19,20].

When lowering the crude protein level in the diet, it is critical that we first ensure that diets are not formulated too far below the lysine requirement (Table 4). Assuming a protein deposition of 150 g/d from 20 to 120 kg, adapting equations from Main et al. (2008) [21] and the National Swine Nutrition Guide (van Heugten, 2010 [22]), the equation: g /SID Lysine:Mcal = 0.000146 × (BW, kg)^2^ - 0.0377 × (BW, kg) + 4.352; describes the SID Lysine:calorie ratio for barrows while; g/SID Lysine:Mcal = -0.00000094 × (BW, kg)^3^ + 0.000306 × (BW, kg)^2^ - 0.0435 × (BW, kg) + 4.414) describes the g SID Lysine:Mcal ratio for gilts (Table 1). This model is relatively similar to the model recently presented by the NRC [23] with the exception that the proposed model above increases lysine concentrations for late finishing pigs by about 0.05 percentage units. A second option for estimating Lysine requirements uses g Lysine/kg gain. A review of the literature indicates that for nursery pigs (< 20 kg) require approximately 19 g of SID lysine/kg of gain, whereas finishing pigs require approximately 20 g/kg of gain. With this approach, accurate growth and energy intake curves are required to generate a customized Lysine:calorie ratio. As an increasing variety of feed ingredients are used, the range of dietary energy levels has expanded increasing the need for accurate Lysine:calorie ratios in diet formulation. The requirements for the other essential amino acids in relation to lysine must also be considered to allow crude protein to be lowered to minimal levels (Table 5).

**Table 4:** Standardized ileal digestible lysine recommendations as influenced by weight

		**Barrows**^**1**^		**Gilts**^**2**^	
**Pig weight, kg**	**g/kg of gain**	**g/Mcal ME**	**%**^**3**^	**g/Mcal ME**	**%**^**3**^
5	19	4.17	1.40	4.20	1.40
10	19	3.99	1.34	4.01	1.34
15	19	3.82	1.28	3.83	1.28
20	19	3.66	1.22	3.66	1.23
30	20	3.35	1.12	3.36	1.13
40	20	3.08	1.03	3.10	1.04
50	20	2.83	0.95	2.89	0.97
60	20	2.62	0.88	2.70	0.91
70	20	2.43	0.81	2.55	0.85
80	20	2.27	0.76	2.41	0.81
90	20	2.14	0.72	2.29	0.77
100	20	2.04	0.68	2.18	0.73
110	20	1.97	0.66	2.08	0.70
120	20	1.93	0.65	1.98	0.66

^1^Barrow lysine concentrations based on the equation: g/SID Lys:Mcal = 0.000146 × (BW, kg)^2^ - 0.0377 × (BW, kg) + 4.352.

^2^Gilt lysine concentrations based on the equation: g/SID Lys:Mcal = -0.00000094 × (BW, kg)^3^ + 0.000306 × (BW, kg)^2^ - 0.0435 × (BW, kg) + 4.414).

^3^Percentages are for diet containing 3,350 kcal ME/kg using NRC [23] nutrient values.

**Table 5:** **Suggested minimum standardized ileal digestible amino acid ratios for growing swine**^**1**^

	**Pig weight range, kg**					
**Amino acid**	**4 to 25**	**25 to 40**	**40 to 60**	**60 to 80**	**80 to 100**	**100 to 130**
Lysine	100	100	100	100	100	100
Threonine^2^	62	61	61	62	63	64
Methionine^3^	28	28	28	28	28	28
Methionine + cysteine^4^	58	56	56	56	57	58
Tryptophan ^5^	18+	18+	18+	18+	18+	18+
Isoleucine^6^	52	52	52	52	52	52
Valine	65	65	65	65	65	65

^1^Adapted from Shannon and Allee, [24] with updates from recent research conducted by the authors and summarized in this paper. Ratios are based on NRC [23] nutrient levels for ingredients. Nutritionists should review their ingredient nutrient values relative to NRC [23] to apply these ratios to their diets.

^2^Threonine:lysine = 0.0000130x^2^ - 0.0014229x + 0.6387290.

^3^Methionine:lysine = 0.0000020x^2^ - 0.0000808x + 0.2806061.

^4^Methionine & Cysteine: lysine = 0.0000113x^2^ - 0.0012621x + 0.5785238.

^5^Tryptopan:lysine ratio appears to be increased when the diet contains large excesses of large neutral amino acids (leucine, isoleucine, valine, phenylalanine, and tyrosine). Improvements in pig growth have been observed with Trp:Lys ratios greater than 18%.

^6^Ratio is at least 60% when high levels of blood meal or cells are included in the diet. Ratio may be lower than 52% when blood cells are not included, but more research is required to verify and to determine the optimal ratio of isoleucine to leucine.

## Threonine:lysine ratio

Deficiencies of threonine result in relatively small reductions in growth and efficiency as compared to deficiencies of the other major amino acids. However, the large difference between apparent and standardized digestibility values for threonine has caused some confusion when setting requirements on a digestible basis. Compared with other amino acids, threonine digestibility increases the most when moving from an apparent to standardized digestibility basis. Van Milgen and Le Bellego [25] conducted a meta-analysis of 22 different studies and found the optimal threonine:lysine ratio increased from 58% at 15 kg to 65% at 110 kg using a linear-plateau model. Use of curvilinear models resulted in higher requirement estimates. In two separate experiments, Lenehan et al. [26] found an optimal threonine:lysine level of 64 to 66% for 10 to 20 kg pigs. James et al. [27] also found the optimal threonine:lysine ratio to be 60 to 65% for 10 to 20 kg pigs. Although Wang et al. [28] did not report a SID threonine:lysine ratio, the growth rate of pigs in their study can be used to estimate the SID lysine requirement (19 g/kg of gain) to calculate SID threonine to be at least 60% of lysine. Based on the above findings, we believe that the threonine requirement can be modeled as a ratio relative to lysine in early growing pig diets (0.0000130BW^2^ - 0.0014229BW + 0.6387290), and like NRC 2012 [23] estimates, increases as the pig becomes heavier (Table 5).

## TSAA:lysine ratio

Considerable research has been conducted in recent years on the total sulfur amino acid requirement and individual requirements for methionine and cysteine. It is generally assumed that methionine must constitute at least 50% of the TSAA ratio (NRC = 48% on weight basis); however, recent data suggests that methionine may need to be slightly greater (55% on weight basis; 50% on molar basis) than cysteine in the ratio [29].

For nursery pigs, Dean et al., [14] suggested that the requirement for total sulfur amino acids was 10.1 g/kg gain or 54% of lysine for 6 to 12 kg pigs. Gaines et al. [26,30] found a slightly higher ratio of 57 to 61% depending on the response criteria and method of assessing the breakpoint with 8 to 26 kg pigs. Yi et al. [31] found a similar TSAA:lysine ratio of 58% for optimal ADG of 12 to 24 kg pigs. In a series of experiments, Schneider et al. [32] found a similar range of SID TSAA:lysine ratios of 57 to 60% for 10 to 20 kg pigs.

## Tryptophan:lysine ratio

Conclusions as to the optimal tryptophan to lysine ratio are difficult to assess for several reasons. Because of the relatively low inclusion rates and small differences in range of tryptophan levels tested (ex. 14 to 22% of lysine), diet manufacturing can be challenging to ensure the low volume test ingredient additions are thoroughly mixed. Also, tryptophan is a difficult amino acid to analyze and different analytical techniques yield different results adding to the confusion. There is also disagreement in the quantity of tryptophan present in key basal ingredients used in many of the research trials, which can dramatically impact the projected ratios because the basal ingredients such as corn make up such a large proportion of the tryptophan in test diets. Finally, the level of other large neutral amino acids in the diet may influence the response to increasing tryptophan levels. The optimal tryptophan:lysine ratio suggested by most studies ranges from 16 to 20%. Although this range is relatively small, the difference can lead to large changes in diet formulation and cost.

On the low end of the recommended range for nursery pigs, Ma et al. [33] suggested that the SID tryptophan:lysine requirement may be as low as 15% for 11 to 22 kg pigs; however, data from Nemechek et al. [34] demonstrates that 15% SID tryptophan:lysine results in lower ADFI and ADG than a ratio of 20%. Guzik et al. [35] estimated the SID tryptophan requirement for nursery pigs at 0.21, 0.20, and 0.18% of the diet for 5.2 to 7.3 kg, 7.3 to 10.2 kg, and 10.3 to 15.7 kg pigs, respectively. Using the SID lysine levels suggested above, these ratios would all be less than 16% of lysine. Jansman et al. [36] found higher estimates for SID tryptophan for 10 to 20 kg pigs, both as a percentage of the diet (0.22%) and as a ratio to lysine (21.5%). In a review of 33 experiments, Susenbeth [37] summarized that the SID tryptophan:lysine requirement is below 17.4% and likely near 16.0%. Susenbeth also concluded that feeding at 17% would include a safety margin to cover most of biological variations and that the tryptophan:lysine ratio seemed to be unaffected by body weight, growth rate, lysine and protein concentration in the diet, or genetic potential of the animals.

**Figure 3:**
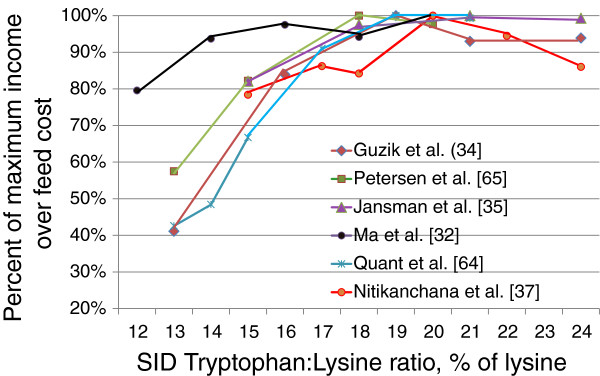
**The effects of increasing tryptophan:lysine ratio on income over feed costs.** Lines represent the change in income over feed cost with increasing standardized ileal digestible tryptophan:lysine ratio from 6 experiments [33,35,36,38,41,42].

Recently Nitikachana et al. [38,39] conducted a series of tryptophan studies in nursery and finishing pigs designed to determine the requirement relative to lysine on an SID basis. They observed that the ideal ratio was no less than 19 to 20% of lysine, which is much greater than previous estimates when evaluated on an economic basis. Furthermore, Slayer et al. [40] also observed a tryptophan requirement of at least 19% of lysine in finishing pigs fed diets containing 30% dried distillers grains with solubles. What is interesting in the pig’s response to tryptophan is that while an optimum “requirement” level can be determined, there is usually a continued, albeit, small improvement in growth performance when feeding levels above the requirement. As a result, from an economic analysis, it is by far safer and, even more economical to be over the estimated requirement than to be below the requirement estimate. This attribute is demonstrated when looking at tryptophan:lysine ratios relative to income over feed cost (IOFC; Figure 3). When the tryptophan:lysine ratio drops below 16% of lysine, profitability decreases dramatically; however, feeding higher ratios in most studies does not decrease IOFC and in some cases increases profitability. When comparing methods to increase the tryptophan:lysine ratio, research suggests that using either added crystalline tryptophan or soybean meal results in similar pig performance [39].

## Valine:lysine ratio

Numerous valine trials have been published in the last 10 years. Mavromichalis et al. [43] was one of the first publications to suggest that the valine requirement of nursery pigs was greater than the level suggested by NRC 1998 [44]. Their data suggested that 10 to 20 kg pigs required 12.5 g of SID valine per kg of gain. Gaines et al. [45] found a similar requirement of 12.3 g of SID valine/kg of gain for 13 to 32 kg pigs. Using the requirement of 19 g of SID lysine per kg of gain for nursery pigs found by several researchers and discussed earlier in this paper, a SID valine:lysine ratio of 66% can be calculated, which is similar to the 65% reported by Gaines et al. [45] for 13 to 32 kg pigs and 65 to 67% reported by Wiltafsky et al. [46] for 8 to 25 kg pigs. A 65% SID valine:lysine ratio was observed by Nemechek et al. [47] using 7 to 12 kg pigs. Nutrient profiles for different ingredients are important when discussing amino acid ratios. For example, a corn-soybean meal based diet formulated to a 65% SID valine:lysine ratio using nutrient values from NRC 1998 [44] will contain 69% SID valine:lysine using values from NRC 2012 [23]. Thus, the ratio used in diet formulation needs to be increased simply due to a change in nutrient profiles.

## Isoleucine:lysine ratio

Similar to other amino acids, our understanding of the optimal ratios of isoleucine to lysine has increased greatly in the last 10 years. The main confusion in understanding the optimal isoleucine to lysine ratio is the interaction between isoleucine and other branch chain amino acids, in particular leucine. Excess leucine in the diet increases branch chain keto acid dehydrogenase levels which lead to catabolism of all branch chain amino acids, further leading to increased requirement for isoleucine due to the increased breakdown.

Spray dried blood cells have been used in several isoleucine studies to create a basal diet with a low isoleucine:lysine ratio [48-51]. The problem with this approach is that blood cells contain high leucine levels, which later were determined to increase the isoleucine:lysine recommendation. Subsequently, Fu et al. [52,53], Dean et al. [54], and Wiltafsky et al. [55] demonstrated that the SID isoleucine:lysine requirement was 60% or greater in diets containing blood meal or blood cells and closer to 50% for diets without high levels of blood cells. The requirement of 50% or less for SID isoleucine:lysine when blood cells are not included in the diet was confirmed by Barea et al. [56] for 11 to 23 kg pigs. Lindemann et al. [57] also found the SID isoleucine:lysine requirement to be between 48 and 52% for ADG. Norgaard and Fernandez [58] found that increasing the isoleucine:lysine ratio from 53 to 62% did not influence performance of 9 to 22 kg pigs. Therefore, it appears that the SID isoleucine:lysine is less than 52% for diets that don’t contain a protein source such as blood products that provide excess leucine in relation to the isoleucine level.

Caution is advised with all branch chain amino acids such as valine, isoleucine, and leucine, as feeding as little as 5% below the minimum ratio (ex. 45 vs 50% of lysine) will greatly reduce feed intake and daily gain. Another concern is that with low-protein amino acid fortified diets formulated to the 5^th^ and 6^th^ limiting amino acid, leucine can become limiting or very near its requirement estimate at 100% of SID lysine [23].

## Nonessential amino acid requirement

Although the order can vary with different dietary ingredient mixtures, typically the first 5 limiting amino acids for most practical diets are lysine, threonine, methionine, tryptophan, and valine. However, formulating diets with high levels of crystalline amino acids to the optimal ratio for the first 5 limiting amino acids often has resulted in poorer performance than diets with high levels of intact protein sources. Kendall et al. [59] found that certain nonessential amino acids (ex. glycine) were required in corn-soybean meal diets with high levels of crystalline lysine and that the nitrogen could not be provided by non-protein nitrogen. In a series of experiments, Powell et al. [60,61] and Southern et al. [62] found that glycine and another amino acid to provide nitrogen were required in diets formulated to the fifth or sixth limiting amino acid in order to maintain feed efficiency.

Another method to ensure that the diet contains enough nonessential amino acids is to place a maximum on the total lysine to total crude protein ratio in diet formulation. The biological basis for a lysine:CP ratio originates from the level of total lysine as a percentage of crude protein in muscle, which ranges from 6.5 to 7.5%. Although an average lysine:CP ratio of 6.8% is often cited, a higher lysine:CP ratio can be used in the diet because the lysine released during normal muscle protein breakdown is conserved and recycled with greater efficiency than other amino acids. Ratliff et al. [63] suggested that the total lysine:CP ratio should not exceed 7.1%. Nemechek et al. [64] found that feed efficiency was only poorer when the total lysine:CP ratio exceeded 7.35%. More research is clearly needed to continue to expand our understanding of nonessential amino acid needs of the pig.

Nonessential amino acids appear to play a particularly important role immediately after weaning due to their high requirement for intestinal growth. For instance, glutamine serves as a primary fuel for the intestinal mucosa. Glutamine and glycine stimulate polyamine synthesis. Arginine is the precursor for polyamines and nitric oxide which is important for regulation of intestinal blood flow and migration of intestinal epithelial cells. Numerous other roles of the nonessential amino acids are reviewed by Wu [65].

## Conclusion

The immune system elicits a variety of responses orchestrated by cytokines. Of these responses, anorexia or reduced energy intake is the limiting factor for protein synthesis. While the amino acid requirements may increase with immune system activation, from a practical standpoint, the decrease in muscle accretion will offset most of the changes in requirements. The evidence is sparse or equivocal for increasing nutrient requirements during an immune challenge. However, some ingredients and diet formulation techniques can help the pig counteract some of the normal gut changes that occur at weaning. Low-protein, amino acid fortified diets can limit the amount of fermentable protein presented to the gut and help reduce post-weaning diarrhea. In these cases, proper amino acid fortification and ratios relative to lysine are essential not to limit pig growth. The ultimate goal for nutritionists is to help the pig transition through this phase without incurring excessive diet cost.

## Competing interests

The authors declare that they have no competing interests.

## Authors’ contributions

BG, MT, SD, JD, and JW were all involved in preparing and contributing to the review. All authors read and approved the final manuscript.
